# Patient- and Clinician-Reported Oral Health Data in Primary Dental Care Practices in Lebanon

**DOI:** 10.7759/cureus.86434

**Published:** 2025-06-20

**Authors:** Dany Daou, Antoine Choufani, Mohamad Mashmouchi, Mounir Doumit

**Affiliations:** 1 Dental Public Health, Lebanese University, Beirut, LBN

**Keywords:** dentistry, health impacts, oral health, oral health observatory, practice-based research

## Abstract

Introduction

The Fédération Dentaire Internationale (FDI) World Dental Federation, in collaboration with the International Consortium for Health Outcomes Measurement (ICHOM), developed a standardized set of oral health outcomes. A study was initiated in 12 countries to validate the data collection tools and methods used in dental practice. The aim of this study was to report the methods used and results collected by the study conducted by the FDI, under the umbrella of the Lebanese Dental Association, in Lebanon.

Methods

Two different questionnaires were administered about the patient’s clinical oral health status - one filled by the patient and one by the dentist.

Results

A total of 798 patients were recruited by 26 dentists. Of these patients, 40.0% reported good oral health, 44.7% reported good general health, 65.5% reported that their last dental visit was within the last year, and 61.4% reported that they brush their teeth ≥2 times/day. The calculated mean oral health impact score was 6.4. More than 30% of patients consumed sugary foods/drinks. The mean number of teeth with caries was 3.7, and the mean number of filled teeth was 6.6. Gingivitis was the most common periodontal disease (40.6%).

Conclusion

The results of this study highlight the poor oral health status among Lebanese citizens, and the situation is expected to deteriorate due to the economic crisis. The National Committee of Oral Health should implement a new strategy to combat this disease and improve oral health status.

## Introduction

Oral health, an aspect of the patient’s overall health that affects the individual’s social, functional, mental, and emotional well-being, often remains unintentionally dismissed [1]. The main oral diseases include dental caries, periodontal disease, edentulism, and oral cancer [2]. While the aforementioned conditions are considered the main oral diseases, the Fédération Dentaire Internationale (FDI) World Dental Federation has defined oral health as a much broader entity encompassing other elements including oral abilities such as chewing, smiling, swallowing, tasting, and speaking; expressive abilities through facial expressions; absence of pain and discomfort; absence of craniofacial complex diseases; physical and mental health; physiological, social, and psychological attributes; and the person’s experiences, perceptions, expectations, and ability to adapt to different situations [3].

Over the last three decades, a huge increase in the number of patients with untreated oral diseases has been observed [1]. In 2019, oral diseases were reported in 46.5% of the population residing in the Eastern Mediterranean Region (330 of 700 million), with a clear disparity between different social classes [2]. In Lebanon, the prevalence of untreated caries of permanent teeth in individuals aged ≥5 years was 34.9% in 2019 [4]. A lower prevalence was observed for severe periodontal disease (≥15 years, 16.7%) and edentulism (≥20 years, 10.0%) [4]. In 2020, the incidence of lip and oral cavity cancer was estimated at 1.1 per 100,000 individuals, with 84 new cases reported that year [4]. 

With a well-structured definition for oral health, the FDI took a further step to standardize collection methods for oral health data. The Oral Health Observatory (OHO) project was thus established by the FDI along with the development of a standardized set of oral health outcomes by the FDI and the International Consortium for Health Outcomes Measurement (ICHOM) [5-7]. A feasibility study was initiated in 12 countries to validate the data collection tools and methods used in routine dental practice.

Lebanon was one of the countries recruited for this study in April 2019, and data collection started in June 2019. The present publication aims to shed light on the oral health data collected by the aforementioned study conducted by the FDI, under the umbrella of the Lebanese Dental Association, among Lebanese citizens with a cut-off date of July 2020, when the whole OHO project was halted due to the COVID-19 pandemic. Previous publications [6,7] have reported on six countries - China, Colombia, India, Italy, Japan, and Lebanon - with advanced data collection as of July 2020.

## Materials and methods

Ethical approval

The OHO project was approved by the Lebanese Dental Association Ethics Review Board (application 54ETH/19). The study was conducted according to national laws and regulations. Patients were well informed about the project prior to their participation in the study. A study information sheet [7] was provided to each participant by their treating dentist. Participants were also required to sign an electronic informed consent form before filling the questionnaire.

Study design and questionnaire development

This study was part of a larger cross-sectional multinational observational study conducted by the FDI, under the umbrella of the Lebanese Dental Association, for patients seeking dental care. Two questionnaires were administered during this study. These questionnaires have been developed by the OHO Task Team to ensure the collection of the most relevant information in the areas of interest, and to ensure alignment with FDI’s new oral health definition. The OHO experts, and experts from FDI’s Vision 2020 project who worked on the FDI oral health definition, agreed on which core elements of oral health should be covered by the survey to best meet the study goals. Participating National Designated Authorities also had the opportunity to review questions and propose changes and additions, considering the relevance and appropriateness of questions to the national context.

The two questionnaires were incorporated in a mobile application and were about the patient’s clinical oral health status; each patient was requested to fill one of the questionnaires, and the treating dentist had to fill the other one (see Appendices). The questionnaires were available in Arabic and French.

Further details on the development of the questionnaire, country recruitment, patient/dentist sampling and recruitment, data collection, patient-reported and dental-reported outcomes have been previously published [6,7].

Dentist sampling and recruitment

A multistage sampling method was used to select dentists. Dentists registered with the Lebanese Dental Association were recruited from the five administrative provinces of Lebanon: Beirut, Mount-Lebanon, Bekaa, North Lebanon, and South Lebanon. The number of dentists recruited in each province was based on the proportion of Lebanese citizens residing in each. A minimum of 24 dentists were required to be enrolled in this study, and each dentist was provided with a guidance document [6] prior to the initiation of the study. After signing their consent and commitment to participate in this study, the FDI provided each recruited dentist with a tablet for filling the questionnaires. Dentists were instructed to follow the guidance document, and a formal training session was held. During this session, each question was explained in detail, and the dentists were informed on how to use the application, perform the data entry, and recruit patients. However, there was no calibration in terms of standard clinical examinations.

Patient sampling and recruitment

Patient selection was based on a modified systematic sampling method. There were no specific inclusion criteria for recruitment - the patient was only required to be able to provide informed consent through the mobile application for their participation in this study and to reside in Lebanon. Informed consent was given by the parents for children aged less than 12 years.

Patients were randomly selected based on the day they visited the clinic and the order of appointments. To further explain this system, on the first day of the study (i.e., Day 1), the patient who arrived first at the clinic was surveyed, and on Day 2, the patient who arrived second at the clinic was surveyed. This process was continued until each dentist surveyed 50 patients [7,8]. In case a selected patient refused to participate in the study, the next patient was surveyed.

Data collection

As previously stated, two questionnaires were filled for each patient’s data. All questionnaires were completed at the dental clinic. Patients completed the patient questionnaire prior to their appointment using the dentist’s tablet, and dentists completed the dentist questionnaire during the patient’s appointment or afterwards. Encrypted data from the mobile application was automatically transferred to the FDI services, and the two datasets, i.e., the patient’s and dentist’s, were linked through participant identification numbers. Data from pediatric patients aged <18 years were collected; however, their results were not included in this analysis. More details on the data collection method have been previously published [7].

Variables

Patient-reported variables included sociodemographic variables (age, sex, and education), self-reported oral health (the Oral Impacts Score and frequency of tooth sensitivity), self-reported general health, and self-reported oral health-related behavior (frequency of brushing teeth, consumption of sugary drinks, and the last dental visitation) (Table 1). Dentist-reported (clinical) variables included the remaining number of teeth, the number of teeth with caries, and periodontal status (Table 1). The English versions of the patient and dentist questionnaires, with a full description of the variables, are found in the Appendices, retrieved from a previous publication [7].

**Table 1:** Patient-Reported and Dentist-Reported Variables of Interest for the Current Analysis

Question	Response Options
Patient-reported variables – Sociodemographic variables	
What is your age?	Open-ended question – Continuous variable – Response in years
What is your sex?	Response options: Female; Male; Rather not answer
What is the highest level of education you have completed or the highest degree you have achieved?	Response options: No formal education; Early childhood education; Primary Education; Lower secondary education; Upper secondary education; Post-secondary non-tertiary education; Short-cycle tertiary education; Bachelor’s or equivalent level; Master’s or equivalent level; Doctoral or equivalent level; Rather not answer Regrouped into: None; Early childhood; Primary level; Secondary level; Degree level; Postgraduate; No answer
Patient-reported variables – Oral health-related behavior	
How often do you brush your teeth or dentures?	Response options: Two or more times per day; Once per day; A few times per week; Once per week; Never; Not applicable; Don’t know/not sure; Rather not answer
Do you use any of the following fluoride products (pick all that apply)?	Response options: Toothpaste; Tablets/drops; Salt; Mouth rinse; Other; Don’t know/not sure; Rather not answer
Do you wear a denture or removable appliance?	Response options: No; I wear a full upper denture; I wear a full lower denture; I wear a partial upper denture; I wear a partial lower denture; I wear an ortho retainer; I wear a mouthguard; I wear a removable orthodontic appliance; Rather not answer
How often do you eat any of the following foods, even in small quantities: Biscuits, cakes, buns, sweets/candy, jam or honey, chewing gum with sugar [plus any locally appropriate options set by NDA]? Seldom/never; Rather not answer	Response options: Four or more times a day; 2-3 times a day; Every day; Several times a week; Once a week; Several times a month;
How often do you eat any of the following foods, even in small quantities: Fresh fruit, fruit and vegetable juice?	Response options: Four or more times a day; 2-3 times a day; Every day; Several times a week; Once a week; Several times a month; Seldom/never; Rather not answer
How often do you drink any of the following beverages, even in small quantities: Tea or coffee with sugar, lemonade, cola, other soft drinks?	Response options: Four or more times a day; 2-3 times a day; Every day; Several times a week; Once a week; Several times a month; Seldom/never; Rather not answer
How often do you have a drink containing alcohol?	Response options: Never; Monthly or less; 2-4 times per month; 2-3 times per week; 4 or more times per week; Rather not answer
When did you last visit the dentist?	Response options: Less than 1 year; 1-2 years; 2-3 years; More than 3 years; Never; Don’t know/Not sure; Rather not answer
Do you have sensitive teeth?	Response options: Often; Occasionally; Rarely; Never; Don’t know/not sure; Rather not answer
Do you consume tobacco products?	Response options: Yes, I smoke cigarettes; Yes, I smoke cigars; Yes, I chew tobacco; Yes, I smoke the shisha (also known as hookah); I used to consume tobacco, but don’t anymore; No, I do not consume tobacco; Rather not answer
Patient-reported variables – Self-reported general health	
How would you rate your general health?	Response options: Very poor; Poor; Fair; Good; Very good; Rather not answer
Dentist-reported variables – Clinical	
How many teeth does the patient have present in his/her mouth?	Open-ended question – Continuous variable
How many teeth with caries does the patient have?	Open-ended question – Continuous variable
How many filled teeth does the patient have?	Open-ended question – Continuous variable
How many missing teeth (out of 32 permanent teeth) does the patient have?	Open-ended question – Continuous variable
Does the patient have acid erosion?	Response options: Yes; No
How would you describe the patient’s periodontal status?	Response options: Healthy; Gingivitis only; Shallow pocket (4-5mm); Deep pocket (>6mm); Mobile teeth

Data analysis

Descriptive statistics were used to determine the numbers and percentages for categorical variables, while mean and standard deviation (SD) were calculated for continuous data.

The Oral Impacts Score was calculated by summing up the individual scores of nine items that are related to impacts associated with the mouth, teeth, or dentures during the past 12 months. The nine items (i.e., problems) are presented in Table 2. Each participant rated the extent to which these items affected them on a scale from 0 to 5, with “0” designating no impact (“not at all”) and 5 designating a high impact (“very much”). In case a participant responded by “No” to any of these items, a score of 0 was allocated to the corresponding item. The overall impact scale ranged from a minimum of 0 to a maximum of 45.

**Table 2:** The Oral Impacts Score and Its 9 Constituent Items

Number	Item
1	Pain
2	Discomfort
3	Spitting or seeing blood when brushing
4	Difficulty eating, chewing or biting food
5	Difficulty speaking or pronouncing words
6	Feeling embarrassed to smile or laugh
7	Problems sleeping
8	Limiting participation in social activities/ difficulty enjoying contact with others
9	Difficulty carrying out work

## Results

Patient demographics

A total of 798 patients were recruited by 26 dentists from five administrative provinces: six dentists from Mount Lebanon and five each from the remaining provinces, Beirut, Bekaa, North Lebanon, and South Lebanon. Adult patients with a mean ± SD age of 38.9 ± 15.2 years participated in this survey. Females and males were almost equally distributed, and the majority of patients had a tertiary education (degree and postgraduate level, n=475, 59.5%) (Table 3). 

**Table 3:** Demographics of Study Participants Abbreviations: n, number of participants; N, total number of participants; SD, standard deviation. Percentages are calculated based on N.

Variable	Study Participants in Lebanon N=798
Age, years	
Mean age (SD)	38.9 (15.2)
Range	18-85
Sex, n (%)	
Male	393 (49.2)
Female	402 (50.4)
Rather not answer	3 (0.4)
Education, n (%)	
None, Early childhood, Primary	77 (9.6)
Secondary level, Post-secondary	243 (30.5)
Degree level (Bachelor, short cycle tertiary)	262 (32.8)
Postgraduate (Masters, Doctoral)	213 (26.7)
Rather not answer	3 (0.4)

Patient-reported oral and general health

Of the 798 patients, 319 (40.0%) and 357 (44.7%) patients reported having good oral health and good general health, respectively. Very poor oral health and very poor general health were reported by 28 (3.5%) and eight (1.0%) patients, respectively. A total of 743 patients (93.1%) considered that oral health has a good impact on the general well-being of the individual, and the calculated mean ± SD Oral Health Impact Score was 6.4 ± 7.1. One third of patients (n=267, 33.5%) considered that their oral problems negatively affected their satisfaction in life (Table 4).

**Table 4:** Self-Reported (Oral) Health and the Impact of Oral Health in Daily Life Abbreviations: n, number of participants; N, total number of participants; SD, standard deviation. Percentages are calculated based on N. The percentages may not sum up to 100% due to rounding.

Variable	Study Participants in Lebanon N=798
Oral health, n (%)	
Very poor	28 (3.5)
Poor	73 (9.1)
Fair	231 (28.9)
Good	319 (40.0)
Very good	147 (18.4)
General health, n (%)	
Very poor	8 (1.0)
Poor	28 (3.5)
Fair	163 (20.4)
Good	357 (44.7)
Very good	241 (30.2)
Rather not answer	1 (0.1)
Life less satisfying due to mouth/teeth problems, n (%)	
Yes	267 (33.5)
No	513 (64.3)
Rather not answer	18 (2.3)
Oral health has a good impact on well-being, n (%)	
Agree/Strongly agree	743 (93.1)
Disagree/Strongly disagree	45 (5.7)
Don’t know	9 (1.1)
Rather not answer	1 (0.1)
Sensitive teeth, n (%)	
Never	250 (31.3)
Rarely	140 (17.5)
Occasionally	148 (18.5)
Often	40 (5.0)
Don’t know	29 (3.6)
Rather not answer	6 (0.8)
Not available	185 (23.2)
Oral health impact score	
Mean (SD)	6.4 (7.1)
Range	0-40

Patient-reported oral health-related behavior

Of the 798 patients, 523 (65.5%) reported that their last dental visit was within the last year, and 10 patients (1.3%) stated that they had never visited a dentist prior to the current visit. The majority of patients reported that they brush their teeth twice or more a day and that they use a fluoride toothpaste (n=490, 61.4% and n=673, 84.3%, respectively). Sugary foods and drinks were consumed daily by 306 (38.3%) and 302 (37.8%) patients, respectively. Less than 10% of patients reported consuming these foods and beverages ≥4 times a day (n=49, 6.1% and n=63, 7.9%, respectively) or at a frequency of once a week or less (n=143, 17.9% and n=120, 15.1%, respectively). More than two-thirds of the patients were non-smokers (n=565, 70.8%), and more than half had never consumed alcohol (n=462, 57.9%) (Table 5).

**Table 5:** Oral Health-Related Behaviors of Study Participants Abbreviations: n, number of participants; N, total number of participants. Percentages are calculated based on N. The percentages may not sum up to 100% due to rounding.

Variable	Study Participants in Lebanon N=798
Last dental visit, n (%)	
Less than 1 year	523 (65.5)
1-2 years	174 (21.8)
2-3 years	35 (4.4)
More than 3 years	52 (6.5)
Never	10 (1.3)
Don’t know/Not sure	4 (0.5)
Toothbrushing frequency, n (%)	
2 or more a day	490 (61.4)
1 or more a day	206 (25.8)
Few times a week	48 (6.0)
Once per week	21 (2.6)
Never	11 (1.4)
Don’t know	2 (0.3)
Rather not answer	4 (0.5)
Not applicable	16 (2.0)
Fluoride toothpaste use, n (%)	
Yes	673 (84.3)
No	125 (15.7)
Denture or removable appliance, n (%)	
Yes	156 (19.5)
No	643 (80.5)
Frequency of sugary foods, n (%)	
4+ times a day	49 (6.1)
2-3 times a day	136 (17.0)
Everyday	306 (38.3)
Several times a week	161 (20.2)
Once a week	64 (8.0)
Several times a month	35 (4.4)
Seldom or never	44 (5.5)
Rather not answer	3 (0.4)
Frequency of sugary drinks, n (%)	
4+ times a day	63 (7.9)
2-3 times a day	200 (25.1)
Everyday	302 (37.8)
Several times a week	112 (14.0)
Once a week	46 (5.8)
Several times a month	20 (2.5)
Seldom or never	54 (6.8)
Rather not answer	1 (0.1)
Frequency of alcohol, n (%)	
4+ times a week	26 (3.3)
2-3 times a week	71 (8.9)
2-4 times a month	95 (11.9)
Monthly or less	133 (16.7)
Never	462 (57.9)
Rather not answer	11 (1.4)
Tobacco consumption (cigarettes), n (%)	
Yes	233 (29.2)
No	565 (70.8)

Dentist-reported patient oral health status

Clinicians reported that the mean ± SD number of teeth with caries was 3.7 ± 4.5, and the mean number of filled teeth was 6.6 ± 5.3. The majority of patients did not have acid erosion (n=619, 77.6%), and less than half of the patients (n=344, 43.1%) had a healthy periodontal status. Gingivitis was reported in 324 patients (40.6%) and mobile teeth in only 10 patients (1.3%) (Table 6).

**Table 6:** Clinician-Reported Oral Health of Study Participants Abbreviations: n, number of participants; N, total number of participants; SD, standard deviation. Percentages are calculated based on N.

Variable	Study Participants in Lebanon N=798
Number of teeth	
Mean (SD)	25.9 (7.0)
Range	0-32
Number of teeth with caries	
Mean (SD)	3.7 (4.5)
Range	0-30
Number of filled teeth	
Mean (SD)	6.6 (5.3)
Range	0-28
Number of missing teeth	
Mean (SD)	5.5 (7.0)
Range	0-32
Acid Erosion, n (%)	
Yes	163 (20.4)
No	619 (77.6)
Missing data	16 (2.0)
Periodontal status, n (%)	
Healthy	344 (43.1)
Gingivitis	324 (40.6)
Shallow pocket	87 (10.9)
Deep pocket	25 (3.1)
Mobile teeth	10 (1.3)
Missing data	8 (1.0)

## Discussion

This paper reports on the oral health data of 798 patients, collected in Lebanon as part of the oral health observatory project conducted in 12 countries. Of the 798 patients, only 523 patients (65.5%) visited the dentist within the last year prior to the current visit, even though 319 patients (40.0%) reported that they have good oral health, and 743 patients (93.1%) considered that oral health impacts general health. This difference between patients' perceptions and their actions highlights that patients may not be fully aware of the consequences of the actions taken to maintain a good oral health status. Furthermore, 10 patients (1.3%) reported that they have never visited a dentist. These findings are different than those reported in previous studies. In previous studies, the percentage of patients who visited the dentist was much lower than that reported in the current study (range, 4.8% to 30%), and a slightly higher percentage never visited a dentist in their lifetime (4.5%) [9-11].

A study conducted by Truppa and colleagues (2019) reported that 26.2% of patients residing in close proximity to International Committee of the Red Cross (ICRC)-supported facilities in three provinces in Lebanon had never visited a healthcare facility (dental or other) or that their last visit was ≥12 months before study interview [12]. These differences may be attributed to the study design and participant selection. The current study was conducted in dental clinics where patients already had a scheduled appointment and, as such, may have a higher tendency to visit a dentist, compared to other studies that collected data from the participants’ residences. In addition, we do not have enough data on the oral health status and behaviors of non-Lebanese individuals residing in Lebanon.

Untreated caries and gingivitis are some of the most common oral diseases, despite being preventable. Preventive strategies include simple techniques such as teeth brushing, which might be hard for patients to implement [13]. In the current study, the mean ± SD number of teeth with caries was 3.7 ± 4.5, and gingivitis was reported in 324 patients (40.6%), highlighting that preventative measures may not be adequately implemented. Indeed, 490 (61.4%) of patients reported brushing their teeth twice or more daily, while others brushed less frequently, and even some patients never brushed their teeth (n=11, 1.4%). Additionally, there were reports of high consumption of sugary food and/or beverages; 491 patients (61.5%) and 565 patients (70.8%) consumed these products at least once daily, respectively. The World Health Organization (WHO) has strongly recommended that the amount of free sugars should be limited to less than 10% of the total daily energy intake. This high sugar consumption affects both oral and general health, leading to caries and obesity [14].

The standardized data collection method allowed the comparison of reported oral health data between different countries from different regions and continents. By July 2020, advanced data from this study were also reported by five other countries: China, Colombia, India, Italy, and Japan. Broomhead and colleagues (2024) reported on data from all six countries and compared the results. Lebanon reported the highest mean teeth with caries (3.7) compared to a range of 1.9 to 2.7 reported by the others. On the other hand, the highest percentage of patients reporting having very good or good general health and the highest oral impact mean score of 6.4 were also reported in Lebanon [7]. Indeed, 93.1% of patients believed that oral health had a good impact on the general well-being of the individual. These contradictory findings are somehow alarming. Is it possible that the patients do not consider having untreated caries as a sign of poor oral health? Do they consider other periodontal diseases as more important, and that only those diseases affect oral health?

Only 267 patients (33.5%) considered that their oral health affected their satisfaction in life. Awareness campaigns may be needed to rectify the misconceptions that people have regarding oral health and associated conditions/diseases. It is important to note that, among the six countries, Lebanon has the smallest geographical area and the smallest population size. As such, any quick action taken, even if with limited resources, might have a greater positive impact on patients' oral behavior than in the other countries. Indeed, the Global Strategy on Oral Health is aiming at achieving a universal oral health insurance coverage accessible to all individuals by the year 2030 [15]. With the results obtained in this study, which was conducted prior to the further decline observed in Lebanon’s economic and political situation, it is unlikely that, by 2030, health insurance coverage will be available for all Lebanese citizens. Different stakeholders, represented by the National Committee of Oral Health, should put forth a new strategy, in line with the Global one, to improve oral health status in Lebanon. 

Data collection in Lebanon started in April 2019, and the cut-off date for data collection was in July 2020. During this time period, several life-changing events occurred in Lebanon, including political instability, the COVID-19 pandemic, and a severe economic crisis. The national currency lost 90% of its value [16], and the high inflation rates affected the expenditure habits of Lebanese citizens [17]. On top of that, the pandemic forced governments to implement confinement periods and, as such, limited the citizens’ mobility. All these events may have affected dental visits, thereby skewing the results. 

Even though this cross-sectional study was part of a larger multinational study conducted under the surveillance of the FDI and Lebanese Dental Association, some limitations may affect the generalizability of the results obtained. The questions were translated from English to the country’s native language (Arabic) and to the second commonly used language (French); however, the translations were not validated, which may raise concerns about cross-language validity. Another limitation is that the lack of calibration in terms of standard clinical examinations may introduce inter-rater variability. Additionally, the sample size of 798 patients may be considered relatively small in comparison to other studies that collected dental care data in Lebanon [9,11,12].

An additional limitation is that this survey was practice-based, i.e., conducted in the dental clinic, where this sample might not be representative of the whole patient population in Lebanon, and results might have been skewed towards a more favorable aspect. For example, a lower percentage of patients will report never visiting a dentist compared to at-home interviews. It is important to highlight that the current study was conducted in Lebanon prior to the Beirut port explosion and the further deterioration of the economic situation [18]. Taking these aforementioned factors into consideration, along with the setting and timing of this study, the results obtained may be very optimistic in comparison to the current situation.

## Conclusions

The findings of this 2019 cross-sectional study, carried out by the FDI in partnership with the Lebanese Dental Association, were deeply concerning. They highlighted the poor state of oral health among people in Lebanon. Given the country’s ongoing economic challenges, it’s likely that the situation has only worsened since then. As such, there is an unprecedented urgency to reactivate the National Committee for Oral Health, which encompasses the Lebanese Dental Association as well as other national organizations. This committee should develop and implement a new comprehensive strategy, aligned with the 2030 WHO vision, that focuses on having better oral health outcomes, such as reducing DMFT (Decayed, Missing, and Filled Teeth) through upstream and preventative methods rather than solely by downstream and curative measures.

## Appendices

**Table 7:** Dentist Questionnaire Source: Broomhead et al. [7], licensed under Creative Commons Attribution 4.0 International (CC BY 4.0).

Question	Options
Does the patient visit your practice on a regular basis (at least once per year)?	Yes No New patient/not applicable
What is the purpose of this visit?	Check-up Routine treatment Emergency treatment Other
How many teeth does the patient have present in his/her mouth?	Continuous
How many teeth with caries does the patient have?	Continuous
How many filled teeth does the patient have?	Continuous
How many missing teeth (out of 32 permanent teeth) does the patient have?	Continuous
Does the patient have any sealants present?	Yes No
How would you describe the patient’s periodontal status?	Healthy Gingivitis only Shallow pocket (4-5mm) Deep pocket (>6mm) Mobile teeth
Does the patient have any other oral diseases or conditions (pick all that apply)?	Craniofacial development abnormalities (e.g. cleft lip and/or palate) Oral cancer/pre-cancer Oral infections Malocclusion
Does the patient have acid erosion?	Yes No

**Table 8:** Patient Questionnaire Source: Broomhead et al. [7], licensed under Creative Commons Attribution 4.0 International (CC BY 4.0).

Question	Options
What is your age?	Continuous
What is your sex?	Male Female Prefer not to say
What is the highest level of education you have completed or the highest degree you have achieved?	No formal education Early childhood education Primary Education Lower secondary education Upper secondary education Post-secondary non-tertiary education Short-cycle tertiary education Bachelor’s or equivalent level Master’s or equivalent level Doctoral or equivalent level Rather not answer
When did you last visit the dentist?	Less than 1 year 1-2 years 2-3 years More than 3 years Never Don’t know/not sure Rather not answer
[If patient has previously visited the dentist] What was the purpose of the visit?	Check-up Routine treatment Emergency treatment Don’t know/not sure Rather not answer
[If patient has not visited the dentist in the past year] What were the reasons for not visiting the dentist sooner (pick all that apply)?	Too busy Dental office too far away Nothing wrong with teeth Afraid/don’t like dentists or dental treatment Dental problems not serious enough Could not get an appointment Lack of or insufficient reimbursement Dentist opening hours inconvenient with personal schedule Other Don’t know/not sure Rather not answer
[If patient has previously visited the dentist] How long does it take to travel to the dental office?	Less than 30 minutes 30 minutes to 1 hour 1 to 3 hours More than 3 hours Don’t know/not sure Rather not answer
[If patient has previously visited the dentist] If you need dental care, you usually get an appointment within…	24 hours 1 to 3 days 3 to 7 days 1 to 3 weeks More than 3 weeks Don’t know/not sure Rather not answer
Do you currently have dental insurance for [current year]?*	Yes No Don’t know/not sure Rather not answer
[If patient has previously visited the dentist] Have you ever had a discussion with a dental professional on any of the following (pick all that apply)?	Sensitivity management Bleeding gums Periodontal disease Denture care No, not necessary given condition Rather not answer
How often do you brush your teeth or dentures?	2 or more times per day 1 time per day A few times per week 1 time per week Never Not applicable Don’t know/not sure Rather not answer
[If patient brushes teeth or dentures] When (pick all that apply)?	Morning before breakfast Morning after breakfast After lunch Before going to sleep Other time of day Don’t know/not sure Rather not answer
Do you use any of the following fluoride products (pick all that apply)?	Toothpaste Tablets/drops Salt Mouth rinse Other Don’t know/not sure Rather not answer
Do you wear a denture or removable appliance?	No I wear a full upper denture I wear a full lower denture I wear a partial upper denture I wear a partial lower denture I wear an ortho retainer I wear a mouthguard I wear a removable orthodontic appliance Rather not answer
[If patient wears a denture or removable appliance] Have you used a fixative for your denture or removable appliance?	No Yes, less than once a month Yes, once a month Yes, once a day Yes, overnight Yes, during the day Yes, more than once a day Rather not answer
[If patient wears a denture or removable appliance] Have you do you use a specialized cleanser for your denture or removable appliance?	Overnight During the day More than once a day Once a day Once a week Once a month Less than once a month Never Rather not answer
How often do you eat any of the following foods, even in small quantities… Biscuits, cakes, buns, sweets/candy, jam or honey, chewing gum with sugar [plus any locally appropriate options set by NDA]?	4 or more times a day 2-3 times a day Every day Several times a week Once a week Several times a month Seldom/never Rather not answer
How often do you eat any of the following foods, even in small quantities… Fresh fruit, fruit, and vegetable juice?	4 or more times a day 2-3 times a day Every day Several times a week Once a week Several times a month Seldom/never Rather not answer
How often do you drink any of the following beverages, even in small quantities… Tea or coffee with sugar, lemonade, cola, other soft drinks?	4 or more times a day 2-3 times a day Every day Several times a week Once a week Several times a month Seldom/never Rather not answer
How often do you have a drink containing alcohol?	Never Monthly or less 2 to 4 times per month 2 to 3 times per week 4+ times per week Rather not answer
[If the patient drinks alcohol] How many alcoholic drinks did you have on a typical day when you were drinking in the last 12 months?	0 drinks 1 to 2 drinks 3 to 4 drinks 5 to 6 drinks 7 to 9 drinks 10 or more drinks Rather not answer
[If the patient drinks alcohol] How often did you have six or more alcoholic drinks on one occasion in the last 12 months?	Never Less than monthly Monthly Weekly Daily or almost daily Rather not answer
Do you consume tobacco products?	Yes, I smoke cigarettes Yes, I smoke cigars Yes, I chew tobacco Yes, I smoke the shisha (also known as hookah) I used to consume tobacco, but don’t anymore No, I do not consume tobacco Rather not answer
[If the patient consumes tobacco products] How often?	Less than 1 time per day Between 1 and 5 times per day Between 5 and 20 times per day More than 20 times per day Don’t know/not sure Rather not answer
How would you rate your general health?	Very poor Poor Fair Good Very good Rather not answer
How would you rate your oral health?	Very poor Poor Fair Good Very good Rather not answer
Do you suffer from sensitive teeth?	Often Occasionally Rarely Never Don’t know/not sure Rather not answer
[If the patient suffers from sensitive teeth] When does this sensitivity occur?	While brushing teeth Due to cold drinks, air or food Due to hot drinks, air or food When touching Due to sweet foods Other Rather not answer
Have you experienced discomfort related to your mouth, teeth or dentures during the past 12 months?^	Yes No Rather not answer
Have you experienced pain related to your mouth, teeth or dentures during the past 12 months?^	Yes No Rather not answer
Do you spit blood or see blood when you brush your teeth?^	Yes No Rather not answer
Have you had difficulty eating food, including difficulty chewing or biting, due to problems with your mouth, teeth or dentures during the past 12 months?^	Yes No Rather not answer
Have you had difficulty speaking or trouble pronouncing words related to your mouth, teeth or dentures during the past 12 months?^	Yes No Rather not answer
Have you been embarrassed to smile or laugh due to problems with your mouth, teeth or dentures during the past 12 months?^	Yes No Rather not answer
Have you had problems sleeping related to your mouth, teeth or dentures during the past 12 months?^	Yes No Rather not answer
Have you limited participation in social activities, or had any difficulty enjoying the contact of other people, due to problems with your mouth, teeth or dentures during the past 12 months?^	Yes No Rather not answer
Have you experienced difficulty carrying out your major work or role due to problems with your mouth, teeth or dentures during the past 12 months?^	Yes No Rather not answer
If you work, have you taken time off because of problems related to your mouth, teeth or dentures in the past 12 months? This does not include time off taken for routine dental check-ups.	Yes No Rather not answer
[If patient has taken time off work] Approximately how many days have you taken off in the last 12 months?	Continuous
Have you ever felt that the appearance of your mouth, teeth or dentures affected your ability to interview for a job?	Yes No Not applicable/never had a job interview Rather not answer
Have you found that life in general was less satisfying due to problems with your mouth, teeth or dentures during the past 12 months?	Yes No Rather not answer
My oral health has a good impact on my general wellbeing	Strongly agree Agree Disagree Strongly Disagree Rather not answer
